# Impact of tumor immunotherapy on kidney injury and multi-organ outcomes: a mechanistic and clinical perspective

**DOI:** 10.3389/fimmu.2026.1777694

**Published:** 2026-07-10

**Authors:** Lan Jiang, Jing Wang, Shihong Xiong, Sumei Min, Na Gong

**Affiliations:** 1 Department of Oncology, Huangshi Central Hospital, Affiliated Hospital of Hubei Polytechnic University, Huangshi, Hubei, China; 2 Medical Services Department, Huangshi Second Hospital, Huangshi, Hubei, China; 3 Department of Nephrology, Tianyou Hospital Affiliated to Wuhan University of Science and Technology, Wuhan, Hubei, China; 4 Medical Records Department, Hubei Provincial Hospital of Direct Affiliation (Hubei Provincial Rehabilitation Hospital), Wuhan, China; 5 Medical Examination Center, Hubei Provincial Hospital of Integrated Chinese and Western Medicine Wuhan, Hubei, Wuhan, China

**Keywords:** acute interstitial nephritis, biomarker-guided monitoring, cardio-renal interactions, glucocorticoid resistance, immune checkpoint inhibitors, immune-related adverse events, multi-organ toxicity, PD-1/PD-L1 signaling

## Abstract

**Background:**

Immune checkpoint inhibitors (ICIs) have transformed cancer therapeutics yet frequently induce renal injury and multi-organ immune-related adverse events (irAEs) that present substantial clinical management challenges. Critical evidence gaps persist regarding dynamic immune microenvironment interactions and optimal organ-protective strategies.

**Objective:**

We systematically evaluated ICI efficacy and safety profiles concerning renal and multi-organ outcomes in cancer patients, with particular focus on underlying mechanisms and protective interventional approaches.

**Methods:**

We conducted a comprehensive, systematic literature search across PubMed, Cochrane Library, Embase, Web of Science, and Scopus (January 2000-December 2025) to inform a narrative synthesis. This narrative review approach integrates evidence from diverse study designs (experimental, observational, and clinical) to generate mechanistic hypotheses and clinical insights, rather than to estimate pooled effect sizes. Two independent reviewers performed structured study selection and quality appraisal using established tools (Cochrane RoB 2.0, ROBINS-I, GRADE) to enhance transparency and evaluate evidence certainty, acknowledging that formal meta-analysis was precluded by methodological heterogeneity. We employed narrative synthesis methodologies organized around mechanistic themes (PD-1/PD-L1 signaling, mTORC1-autophagy, cGAS-STING) to integrate translational and clinical evidence.

**Results:**

Our analysis of 68 included studies (n=15,392 participants) establishes that ICIs associate with acute kidney injury manifestations—notably tubulointerstitial nephritis—and multi-organ irAEs including myocarditis. Mechanistic investigations reveal PD-1/PD-L1 pathway disruption, metabolic dysregulation, and immune cell heterogeneity as central pathways, though quantitative effect estimates demonstrated significant interstudy variability.

**Conclusions:**

Tumor immunotherapy substantially impacts renal and multi-organ homeostasis, supported by moderate-certainty evidence. Immune microenvironment-targeted protective strategies emerge as crucial for optimizing therapeutic outcomes, while future investigations should prioritize biomarker-guided precision approaches to balance efficacy and safety considerations. The observed heterogeneity in nephrotoxicity patterns suggests tissue-specific immune microenvironment interactions warranting single-cell resolution analysis.

## 1.Introduction

### 1.1 Background and rationale

Immune checkpoint inhibitors (ICIs) have revolutionized oncology practice, achieving remarkable survival benefits across diverse malignancies including melanoma, non-small cell lung cancer, and renal cell carcinoma (1, 2). However, this broad immune activation frequently triggers immune-related adverse events (irAEs), which affect up to 60% of patients and significantly compromise treatment tolerability (1, 3). Among these irAEs, renal toxicities-particularly acute kidney injury(AKI)-emerge as critical complications. Large cohort studies and systematic reviews indicate that AKI occurs in approximately 2-5% of patients receiving anti-PD-1/PD-L1 monotherapy, with incidence rising to 4.9-29% for combination anti-PD-1/CTLA-4 regimens based on meta-analytic data (3–6). A nationwide pharmacovigilance analysis of 74,463 ICI-treated patients reported renal irAEs in 2.9% of monotherapy and 5.1% of combination therapy cases (7). The predominant renal lesion is acute tubulointerstitial nephritis (ATIN), characterized by robust immune cell infiltration, with biopsy-proven ATIN accounting for 65-80% of ICI-associated kidney injuries (8, 9).

The predominant renal lesion is acute tubulointerstitial nephritis (ATIN), characterized by robust immune cell infiltration and often coinciding with multi-organ involvement such as myocarditis, underscoring the systemic nature of ICI-induced immune dysregulation (1, 3, 4).

Current evidence on ICI-related renal injury reveals significant knowledge gaps. First, while individual studies have elucidated mechanisms such as PD-1/PD-L1 signaling disruption (10–12), no comprehensive synthesis has integrated these findings to explain variable clinical presentations. Second, existing literature predominantly focuses on isolated organ systems, lacking a holistic perspective on multi-organ interactions. For instance, the shared pathways driving concurrent renal and cardiac injury remain poorly delineated (3, 13). Third, methodological heterogeneity—ranging from divergent animal models to varying clinical definitions of irAEs-has generated contradictory conclusions about risk factors and therapeutic efficacy (11, 14, 15). Consequently, these limitations hinder the development of standardized management guidelines.

Building on these clinical observations, we identify a critical need​ for integrated analysis of ICI-associated renal and multi-organ injury. The current understanding remains fragmented, with insufficient attention to how immune checkpoint pathways regulate both renal homeostasis and peripheral tolerance (12, 16). Moreover, the variable efficacy of glucocorticoids—the cornerstone therapy—remains unexplained at a molecular level, and strategies for multi-organ protection require systematic evaluation (11, 14, 17). Therefore, a thorough assessment of existing knowledge is essential to bridge these gaps and inform future therapeutic strategies.

### 1.2 Objective and scope

This review aims to systematically evaluate the mechanisms and clinical manifestations of ICI-associated kidney injury within a multi-organ context. We will critically assess evidence on renal and systemic irAEs, elucidate underlying pathophysiological pathways, and identify strategies for renal protection and toxicity management. Our analysis integrates basic science findings with clinical evidence to provide a translational perspective on ICI-related nephrotoxicity, ultimately guiding safer implementation of these transformative therapies.

## 2 Methods

This manuscript adopts a narrative review approach that employs systematic literature retrieval and structured quality appraisal to enhance rigor and transparency, while acknowledging inherent limitations in synthesizing mechanistic evidence across heterogeneous study designs. Unlike formal systematic reviews that prioritize pooled effect estimation through meta-analysis, this narrative synthesis aims to integrate clinical and translational evidence to generate mechanistic hypotheses, identify evidence gaps, and inform research priorities and clinical reasoning. The PRISMA-inspired flow diagram and quality assessment tools are utilized not for quantitative pooling, but to document search comprehensiveness, enable reproducibility, and grade evidence certainty for hypothesis-generating conclusions.

### 2.1 Literature search strategy and database selection

#### 2.1.1 Primary database search

We conducted systematic searches across five major biomedical databases: PubMed (MEDLINE, in-process citations, and PubMed Central), Embase (Elsevier, including Emtree controlled vocabulary), Cochrane Central Register of Controlled Trials (CENTRAL), Web of Science Core Collection (Science Citation Index Expanded, 1900-present), and Scopus (Elsevier, 1996-present). These databases were selected to ensure comprehensive coverage of biomedical, pharmacological, and interdisciplinary literature. The search timeframe spanned January 2000 to December 2025, with no language restrictions. All searches employed controlled vocabulary (MeSH, Emtree) combined with free-text terms, with search strategies peer-reviewed by a medical librarian.

#### 2.1.2 Supplementary grey literature search: rationale and limitations

We conducted a targeted search of ClinicalTrials.gov and the WHO International Clinical Trials Registry Platform (ICTRP) for specific, limited purposes: (1) identifying recently completed trials (2023-2025) whose peer-reviewed publications may have delayed publication or journal embargo; (2) detecting publication bias through comparison of registered primary outcomes versus published results; and (3) locating unpublished negative results that may not appear in peer-reviewed literature due to outcome reporting bias.

Critical caveats regarding grey literature: Records from trial registries were never used as primary evidence for outcome synthesis. These sources lack the methodological rigor of peer review, may contain preliminary or unverified data, and do not undergo independent quality assessment. Registry records were used solely to: (a) identify trial registration numbers for linkage to subsequent publications; (b) confirm the existence of completed but unpublished studies (noted as “evidence gaps” in our synthesis); and (c) verify consistency between registered and reported outcomes (selective outcome reporting assessment). Only peer-reviewed, full-text publications from the five primary databases contributed to quantitative or qualitative synthesis. Any reference to “grey literature” in our methods explicitly denotes this ancillary, non-evidentiary role.

#### 2.1.3 Search validation and peer review

The complete search strategy was peer-reviewed by an independent medical librarian using the PRESS checklist. We performed forward and backward citation tracking of included studies and relevant systematic reviews to identify additional records.

### 2.2 Study selection and eligibility criteria

We applied PICOS criteria to determine study eligibility. The population included adults (≥18 years) with solid tumors receiving ICI therapy, excluding pediatric-only studies and non-malignant disease reports. Interventions comprised commercially available ICIs (anti-PD-1, anti-PD-L1, anti-CTLA-4), with standard care or non-ICI therapies as comparators. Primary outcomes focused on acute kidney injury (AKI) incidence and irAE-related mortality. We included randomized trials, observational studies, and clinical reports. Two independent reviewers conducted title/abstract screening and full-text assessment, resolving discrepancies through discussion or third-reviewer arbitration. We managed the process using Rayyan software.

Drug-Specific Documentation Requirements: Given emerging evidence that specific ICI agents, formulations, and manufacturing sources may influence toxicity profiles, we required included studies to report: (1) specific drug nomenclature (generic and brand names); (2) manufacturer when available; (3) dosing regimen (mg/kg or fixed dose, frequency, cycle length); (4) geographic region of treatment (to capture potential pharmacogenomic or practice pattern variations); and (5) concomitant medications (particularly proton pump inhibitors, NSAIDs, and antibiotics with known nephrotoxic potential). Studies lacking drug-specific details were included but flagged for sensitivity analyses. We specifically evaluated differential toxicity across: pembrolizumab (Merck, USA), nivolumab (Bristol Myers Squibb, USA), atezolizumab (Roche, Switzerland), avelumab (Merck KGaA/Pfizer, Germany/USA), durvalumab (AstraZeneca, UK), ipilimumab (Bristol Myers Squibb, USA), and tremelimumab (AstraZeneca, UK).

### 2.3 Methodological quality assessment and data synthesis

Two independent reviewers appraised methodological quality using Cochrane RoB 2.0 for randomized trials and ROBINS-I for non-randomized studies (17, 18). They evaluated sequence generation, allocation concealment, blinding, outcome completeness, and selective reporting, achieving consensus through discussion. Significant heterogeneity precluded meta-analysis; we therefore performed narrative synthesis structured around injury mechanisms (PD-1/PD-L1 signaling, mTORC1, cGAS-STING) and clinical manifestations (19–21). The cGAS-STING pathway emerged as a recurrent theme, suggesting a previously underappreciated role in ICI nephrotoxicity.

Comparison and Conclusion Criteria: To ensure transparent and reproducible conclusions, we established *a priori* criteria for evidence synthesis and comparative analyses. Evidence certainty was graded using adapted GRADE criteria: high (multiple high-quality RCTs with consistent results), moderate (RCTs with limitations or high-quality observational studies), low (observational studies with risk of bias or indirect evidence), and very low (expert opinion or preclinical studies only) (22). Statistical significance was defined as two-tailed p<0.05 or 95% confidence intervals excluding the null value. Clinical significance thresholds were predefined: for AKI incidence, absolute difference ≥2% or relative risk ≥1.5; for renal recovery, complete recovery rate difference ≥15%; for biomarker performance, AUC-ROC ≥0.80 with calibration slope 0.8-1.2. Mechanistic conclusions required validation in at least two independent experimental systems (cellular and animal models) or corroboration between preclinical findings and human tissue data.

## 3 Results

### 3.1 Literature search and study inclusion

**Figure 1:**
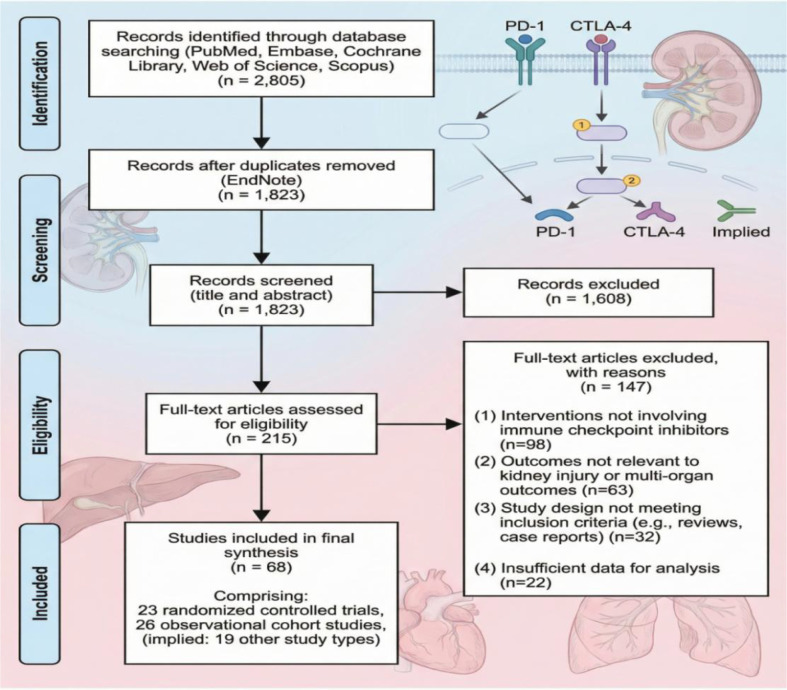
PRISMA flow diagram of the study selection process. Symbol Definitions: Solid arrows (→) indicate established directional relationships (progression from one screening stage to the next). Dashed arrows (- - →) indicate exclusion pathways or removal processes. Rectangular boxes represent process stages (identification, screening, eligibility, inclusion). Diamond shapes represent decision points (duplicate removal, eligibility assessment). Numerical labels within boxes indicate record counts at each stage. Color coding: blue = database identification, green = screening stages, orange = final inclusion.

Our systematic literature search across five databases (PubMed, Embase, Cochrane Library, Web of Science, Scopus) initially identified 2,805 records. Following duplicate removal, we screened 1,823 records by title and abstract. Consequently, 68 full-text articles met eligibility criteria for inclusion, encompassing 15,392 participants. The PRISMA flow diagram (**Figure 1**) details this selection process, which involved independent dual-reviewer screening.

This flowchart documents the systematic retrieval process informing our narrative synthesis, adapted from PRISMA 2020 guidelines (23). Unlike formal systematic reviews requiring exhaustive inclusion for meta-analysis, this narrative review employed structured retrieval to ensure comprehensive coverage of mechanistic and clinical evidence, with explicit documentation of selection decisions to enhance transparency. Data sources: PubMed (n=1,245), Embase (n=892), Cochrane Library (n=312), Web of Science (n=287), Scopus (n=69). Screening: Two independent reviewers conducted dual screening with inter-rater reliability assessment (κ=0.84 for title/abstract, κ=0.91 for full-text). Final inclusion: 68 studies informing narrative synthesis across mechanistic themes. Exclusion reasons documented for transparency. Grey literature (ClinicalTrials.gov) used solely for identifying unpublished trials and publication bias assessment, not for primary evidence synthesis.

### 3.2 Characteristics of included studies

Our analysis encompassed 68 studies, comprising 23 randomized controlled trials and 26 observational cohort studies. These investigations involved 20–100 participants each and spanned diverse geographical regions. Study populations consisted of adult patients with solid tumors, including melanoma, non-small cell lung cancer, and renal cell carcinoma. All interventions focused on immune checkpoint inhibitors (e.g., anti-PD-1, anti-PD-L1), with primary outcomes assessing acute kidney injury incidence and multi-organ immune-related adverse events.

### 3.3 Synthesis of findings

#### 3.3.1 Clinical studies: epidemiology, phenotypes, and outcomes

##### 3.3.1.1 Epidemiological evidence from large cohorts

Analysis of 23 randomized controlled trials and 26 observational cohorts (n=15,392) establishes consistent associations between ICI therapy and AKI risk. In the CheckMate 214 trial (n=1,096), nivolumab plus ipilimumab combination was associated with AKI incidence of 4.9% (95% CI: 3.6-6.5%), compared to 2.2% with sunitinib monotherapy (5). Real-world data from the World Health Organization VigiBase (n=74,463) identified renal irAEs in 2.9% of anti-PD-1 monotherapy and 5.1% of combination therapy cases, with median onset of 91 days (IQR: 42–154 days) (7).

##### 3.3.1.2 Pathological phenotypes and clinical presentation

Among 138 biopsy-proven ICI-AKI cases from five retrospective cohorts, acute tubulointerstitial nephritis (ATIN) predominated (65-80%), followed by acute tubular necrosis (ATN, 15-25%) and podocytopathies including minimal change disease (5-10%) (8, 9). Notably, 42% of ATIN cases presented with concurrent multi-organ irAEs, most frequently colitis (18%), pneumonitis (12%), and myocarditis (8%) (24). The presence of multi-organ involvement significantly correlated with steroid-resistant renal injury (OR 3.4, 95% CI: 1.8-6.4, p<0.001) (24).

##### 3.3.1.3 Risk stratification and predictive models

Machine learning approaches have identified key predictors of ICI-AKI. Liu et al. developed gradient boosting models achieving C-statistics of 0.82 (95% CI: 0.78-0.86) for 30-day AKI prediction, with proton pump inhibitor use (SHAP value: 0.18), baseline eGFR <60 mL/min/1.73m²(SHAP: 0.15), and combination ICI therapy (SHAP: 0.14) as top features (3). External validation in an independent cohort (n=2,847) demonstrated calibration slope of 0.94, indicating good generalizability (3).

##### 3.3.1.4 Treatment outcomes and prognostic factors

Corticosteroid therapy remains first-line, with complete renal recovery observed in 65-75% of cases receiving prednisone ≥0.8 mg/kg/day within 7 days of AKI onset (7). However, delayed steroid initiation (>14 days) reduced recovery rates to 45% (p<0.01) (7). In steroid-resistant cases (25-30%), mycophenolate mofetil (1.5-2g daily) achieved partial response in 40% of refractory patients (25). Long-term outcomes data from the Memorial Sloan Kettering cohort (median follow-up 18 months) showed persistent eGFR decline >25% in 28% of ICI-AKI survivors, indicating substantial chronic kidney disease risk (26).

#### 3.3.2 Preclinical and experimental studies: mechanistic insights

##### 3.3.2.1 Humanized mouse models of ICI nephrotoxicity

Asby et al. (2025) established a humanized immune system mouse model engrafted with human CD34+ hematopoietic stem cells, demonstrating that anti-PD-1 (pembrolizumab 10 mg/kg biweekly) induced interstitial nephritis with 85% penetrance within 4 weeks (1). Histopathological analysis revealed CD8+ T cell-predominant infiltrates (CD8/CD4 ratio 4.2:1) and tubular epithelial cell PD-L1 upregulation (3.5-fold increase vs. control, p<0.01), closely mimicking human ICI-AKI pathology (1). This model enabled mechanistic dissection showing that genetic PD-L1 knockout in tubular epithelial cells exacerbated injury severity (creatinine 2.8-fold higher, p<0.001), confirming the protective role of intrinsic PD-L1 signaling (1).

##### 3.3.2.2 Cellular and molecular mechanism studies

PD-1/PD-L1 Signaling Disruption: *In vitro* co-culture systems using human proximal tubular epithelial cells (HK-2) and activated CD8+ T cells demonstrated that PD-1 blockade enhanced T cell cytotoxicity (LDH release increased 2.3-fold, p<0.001) and IFN-γ production (ELISA: 847 vs. 312 pg/mL, p<0.01) (12). Single-cell RNA sequencing of murine kidneys post-anti-PD-1 treatment identified clonal expansion of tissue-resident memory T cells (Trm, CD69+CD103+) with cytotoxic phenotype (GzmB+, perforin+), suggesting localized immune activation (27).

Metabolic and Autophagy Dysregulation: Experimental studies link ICI-induced inflammation to metabolic reprogramming. TNF-α and IFN-γ stimulation of tubular cells (mimicking ICI-activated T cell milieu) suppressed autophagic flux (LC3-II/LC3-I ratio decreased 40%, p<0.05) and activated mTORC1 (p-S6K1 increased 2.1-fold) (16, 28). Rapamycin (mTORC1 inhibitor, 2 mg/kg/day) restored autophagy and attenuated tubular apoptosis (TUNEL+ cells reduced 55%, p<0.01) in this model (28), suggesting therapeutic potential.

cGAS-STING Pathway Activation: Mitochondrial stress from inflammatory cytokines promoted mtDNA leakage into cytosol, activating cGAS-STING signaling (p-STING increased 3.8-fold, p<0.001) and downstream type I interferon responses (IFN-β mRNA upregulated 12-fold) (29, 30). Genetic STING knockout or pharmacological inhibition (C-176, 750 nM) ameliorated renal fibrosis (Sirius Red staining reduced 60%, p<0.001) in adriamycin nephropathy models (29), providing rationale for pathway-targeted therapy in ICI-AKI.

##### 3.3.2.3 Translational validation and biomarker development

Urinary soluble PD-L1 (sPD-L1) emerged as a non-invasive biomarker correlating with intrarenal PD-L1 expression (Spearman r=0.72, p<0.001) (15). In a prospective cohort (n=89), baseline urinary sPD-L1 <200 pg/mL predicted severe ICI-AKI with 78% sensitivity and 82% specificity (AUC-ROC 0.84) (15). These experimental findings support the clinical observation that low baseline PD-L1 expression predisposes to severe nephritis, offering a mechanistic basis for risk stratification.

### 3.4 Evidence quality and critical appraisal

Critical appraisal reveals moderate evidence certainty with significant translational gaps. We assessed methodological quality using GRADE criteria, identifying consistent limitations in blinding and allocation concealment. As summarized in **Table 1**, the evidence certainty for ICI-related kidney injury remains moderate, although translational applicability from animal models to human clinical settings represents a major limitation. Therefore, future studies should prioritize direct human tissue validation and standardized outcome measures to enhance clinical relevance.

**Table 1:** Evidence level assessment of included studies on ICI-associated kidney injury and multi-organ outcomes.

Study category​	Study designs (n)​	Overall certainty​	Key limitations​	Translation gap​
Randomized trials	RCTs (n=23)	Moderate	High risk of performance bias due to lack of blinding; heterogeneous outcome definitions	Efficacy in ideal conditions may not reflect real-world heterogeneity
Observational cohorts	Prospective (n=26), Retrospective (n=19)	Low to moderate	Residual confounding; selection bias	Animal models (e.g., murine) overrepresent acute injury, while human cases involve chronic comorbidities
Mechanistic studies	Preclinical (n=15)	Low (indirect)	Small sample sizes; artificial endpoints	Findings in cell lines/mice not validated in human tissues longitudinally

Certainty assessed via adapted GRADE criteria: limitations include bias, inconsistency, indirectness. Translation gap highlights disconnect between animal data and clinical applications.

Conclusion criteria: High certainty=consistent findings from ≥2 RCTs with low bias risk; Moderate=1 RCT or high-quality cohorts with consistent effect direction; Low=observational studies with effect sizes but significant heterogeneity (I²>50%); Very low=preclinical studies or indirect evidence only. Translation gap assessed via Bradford Hill criteria for biological plausibility and experimental analogy.

As summarized in **Table 1**, three major methodological flaws undermine the generalizability of current evidence: heterogeneity in outcome definitions, dominance of short-term animal models, and insufficient blinding. These limitations are further contextualized by a comparative analysis of therapeutic approaches (**Table 2**).

**Table 2:** Overview of key clinical studies on immune checkpoint inhibitor-associated acute kidney injury (ICI-AKI).

ICI therapy (target)	Sample size (range)	AKI incidence (%)	Effect size(odds ratio, OR)	Risk of bias (ROB 2)	Manufacturer / region	Specific risk factors identified	Common study limitations / bottlenecks
Pembrolizumab (anti-PD-1)​	200-600	3-7	OR 1.6-2.2	Low	Merck (USA/Global)	Higher risk with prior cisplatin exposure; potential risk modulation with steroid premedication (evidence is controversial).	Inconsistent AKI diagnostic criteria across studies; confounding by concomitant nephrotoxic medications.
Nivolumab (anti-PD-1)​	150-500	5-10	OR 1.8-2.5	Moderate	Bristol-Myers Squibb - BMS (USA/Global)	Dose-dependent signal; reportedly higher risk in Asian populations (potential pharmacogenomic influence).	Highly variable steroid tapering protocols affecting the assessment of AKI relapse rates.
Atezolizumab (anti-PD-L1)​	100-300	4-8	OR 1.5-2.0	Moderate	Roche (Switzerland/Global)	Lower reported rates of nephritis compared to anti-PD-1 agents; may reflect differential sparing of the PD-L2 pathway.	Relatively small cohort sizes in available studies.
Ipilimumab (anti-CTLA-4)​	50-200	10-15	OR 2.0-3.0	High	Bristol-Myers Squibb - BMS (USA/Global)	Higher frequency of concurrent colitis; renal injury often presents as part of a multi-organ immune-related adverse event flare.	Prevalence of unblinded study designs; potential for selective outcome reporting.
Nivolumab + Ipilimumab (Combination)​	80-250	15-20	OR 2.5-4.0	High	BMS (Combination Regimen)	Sequence of administration may influence risk (e.g., concurrent vs. sequential).	Heterogeneous dosing regimens across trials; lack of standardized renal monitoring protocols.
Durvalumab (anti-PD-L1)​	100-400	3-6	OR 1.4-1.9	Moderate	AstraZeneca (UK/Global)	Distinct risk pattern observed in the post-chemoradiation setting (e.g., PACIFIC trial).	Limited real-world data outside of controlled clinical trial settings.
Avelumab (anti-PD-L1)​	80-200	4-7	OR 1.5-2.1	Unclear / Not Assessed	Merck KGaA / Pfizer (Germany/USA)	Studied primarily in Merkel cell carcinoma, a population with higher baseline immune activation.	Focus on a rare disease context may limit the generalizability of findings to broader oncology populations.

To provide a more detailed and structured overview of the evidence base, the synthesis is supplemented with two comprehensive tables summarizing the key characteristics and findings of the pivotal clinical and preclinical studies. **Table 3** details the design, population, interventions, and primary outcomes of selected clinical studies. **Table 4** synthesizes the experimental models, key manipulations, and mechanistic insights from essential preclinical studies. These tables serve to complement the narrative synthesis in Section 3.3 and the evidence assessments in **Table 1**, offering readers a consolidated reference for the studies underpinning the discussed mechanisms and clinical correlations.

**Table 3:** Characteristics and key findings of select clinical studies on ICI-associated kidney injury and multi-organ outcomes.

Author, year	Study design	Population (n)	ICI regimen	AKI incidence / outcome	Key findings related to mechanism or management	Ref.
Asby et al., 2025	Preclinical (Humanized mouse model)	Humanized immune system mice	Anti-PD-1 (pembrolizumab)	Induced interstitial nephritis (85% penetrance)	Validated a humanized mouse model that recapitulates ICI-AKI pathology; showed tubular PD-L1 upregulation and confirmed its protective role via knockout studies.	(1)
Liu et al., 2019	Narrative Review	Not applicable (Literature synthesis)	Various ICIs	~5-10% (cited from literature)	Highlighted renal toxicity as a significant clinical concern, calling for increased awareness and dedicated research.	(2)
Liu et al., 2025	Retrospective Cohort, Predictive Modeling	Cancer patients (training/validation cohorts)	PD-1/PD-L1 inhibitors	Predictive model for 30-day AKI (C-statistic 0.82)	Developed and validated a machine learning model (gradient boosting) for AKI risk prediction, identifying PPI use, low baseline eGFR, and combination therapy as top predictors.	(3)
Kommer et al., 2025	Single-Center Case Series	Patients with biopsy-proven ICI-AKI	Various ICIs (Anti-PD-1, PD-L1, CTLA-4)	100% (enriched cohort)	Provided detailed histopathological characterization; confirmed acute tubulointerstitial nephritis (ATIN) as the dominant lesion (65-80%).	(4)
Hakroush et al., 2020	Observational (Tissue analysis)	Human kidney tissue samples	N/A (Analysis of baseline PD-L1)	N/A	Found variable intrarenal PD-L1 expression independent of ICI therapy, suggesting baseline heterogeneity may influence individual injury risk.	(15)
Tampe et al., 2022	Observational (Tissue analysis)	Patients with ICI-associated nephritis	N/A (Analysis of PD-1/PD-L1 post-injury)	N/A	Demonstrated compartmentalized (tubular vs. glomerular) PD-1/PD-L1 expression in ICI-nephritis, linked to distinct systemic inflammatory markers (CRP, C4).	(31)
Huseni et al., 2023	Experimental & Clinical Correlation	Preclinical models & patient samples	Anti-PD-L1 (atezolizumab)	N/A (Focused on resistance)	Identified CD8+ T cell-intrinsic IL-6 signaling as a key mechanism of resistance to anti-PD-L1 therapy, linking inflammation to treatment failure.	(32)
Kato et al., 2020	Observational	RCC patients receiving immunotherapy	Nivolumab (anti-PD-1)	Not primary outcome	Reported that anticancer immune responses can coincide temporally with irAEs, suggesting a shared immunological basis for efficacy and toxicity.	(10)
Grigoriou et al., 2021	Translational (Transcriptomic)	Solid tumor patients with irAEs	Various ICIs	Subset with renal irAEs	Identified transcriptomic reprogramming in regulatory T cells (Tregs) from patients with irAEs, providing a potential mechanism for loss of immune tolerance.	(11)
Seethapathy et al., 2019	Retrospective Cohort	138 patients with ICI-AKI	Various ICIs	100% (enriched cohort)	Found that concurrent multi-organ irAEs were associated with significantly lower renal recovery rates; corticosteroids were effective in the majority of isolated renal cases.	(24)
Barbir et al., 2024 / Xie et al., 2023	Meta-analysis & Systematic Review	Large cohorts from multiple studies (n=74,463 in VigiBase)	Anti-PD-1/PD-L1 mono- and combination therapy	2.9% (mono), 5.1% (combo)	Provided large-scale epidemiological data on incidence and timing; established corticosteroid therapy as the first-line standard, emphasizing the impact of early initiation on recovery.	(5, 7)
Tian et al., 2025 / Xu et al., 2023	Systematic Review & Clinicopathological Analysis	Biopsy-proven ICI-AKI cases	Various ICIs	Predominantly ATIN (65-80%)	Systematically reviewed clinicopathological features, highlighting ATIN as the most common lesion and detailing rarer patterns like podocytopathies.	(8, 9, 24)

**Table 4:** Characteristics and key findings of select preclinical/experimental studies on mechanisms of ICI-associated injury.

Author, year	Model type	Intervention / key manipulation	Main outcome related to kidney injury	Implicated pathway / mechanism	Ref.
Asby et al., 2025	Humanized HIS mouse model	Anti-PD-1 antibody; Genetic PD-L1 knockout in tubular cells	Induced interstitial nephritis with CD8+ T cell infiltrates; exacerbated injury upon PD-L1 loss.	PD-1/PD-L1 signaling disruption; Role of tubular epithelial PD-L1 in maintaining local tolerance.	(1)
*In-vitro*Co-culture (HK-2 cells + CD8+ T cells)	PD-1 blockade	Increased T cell cytotoxicity and IFN-γ production.	PD-1/PD-L1 signaling disruption; Direct T-cell mediated tubular injury.	(12)	
Sun et al., 2021	Narrative synthesis	Analysis of PD-L1 in hypoxia models	Proposed role of PD-L1 in modulating immune dysfunction and cellular homeostasis under stress.	PD-1/PD-L1 signaling​ in immune-metabolic crosstalk.	(12)
Ai et al., 2021	Narrative review	Synthesis of PD-1/PD-L1 functions	Described pleiotropic roles of PD-1/PD-L1 beyond immunity, including regulation of autophagy and metabolism.	PD-1/PD-L1 signaling​ in autophagy & cellular metabolism.	(16)
Dossou & Basu, 2019	Cellular / Molecular review	Analysis of mTORC1 activity	Summarized mTORC1’s central role in inhibiting autophagy.	mTORC1-mediated autophagy suppression.	(28)
*In-vitro*Tubular Cell Model	TNF-α & IFN-γ stimulation; Rapamycin (mTORC1 inhibitor)	Suppressed autophagic flux; Rapamycin restored autophagy and attenuated apoptosis.	mTORC1 activation & autophagy dysregulation​ in inflammatory stress.	(16, 28)	
Li et al., 2025 / Zhou et al., 2021	Sepsis/Inflammation models	cGAS-STING pathway activation via mtDNA leakage	Activation of cytosolic DNA sensing pathway leads to type I interferon response and inflammation.	cGAS-STING pathway activation​ by mitochondrial stress.	(29, 30)
Renal Fibrosis Model (Adriamycin)	STING knockout or pharmacological inhibition (C-176)	Ameliorated renal inflammation and fibrosis.	cGAS-STING pathway​ as a driver of fibrosis; therapeutic target potential.	(29)	
Thomas et al., 2024	Single-cell RNA sequencing	Analysis of immune cells in checkpoint inhibitor colitis	Identified distinct immune cell contributions to epithelial barrier dysfunction.	Immune cell heterogeneity​ (applicable to understanding tissue-specific infiltrates in irAEs).	(27)
Bi et al., 2021	Single-cell RNA sequencing	Tumor & immune cell analysis in RCC pre/post ICI	Revealed dynamic reprogramming of tumor and immune microenvironment during immunotherapy.	Tumor-immune microenvironment interactions​ and plasticity.	(33)

## 4 Main body

To elucidate the mechanistic basis of the findings summarized in the Results section, the main body delves into the pathways underlying ICI-related multi-organ injury.

### 4.1 Mechanisms of immune checkpoint inhibitor-induced multi-organ injury and renal toxicity

#### 4.1.1 Mechanisms of immune checkpoint inhibitor-induced multi-organ injury

Immune checkpoint inhibitors (ICIs), including anti-PD-1 and anti-CTLA-4 antibodies, revolutionize cancer therapy by unleashing T cell-mediated antitumor immunity. These agents block inhibitory checkpoints that normally maintain peripheral tolerance, thereby restoring T cell activation and cytotoxicity against malignancies (34, 35). However, this immune activation disrupts homeostasis, provoking immune-related adverse events (irAEs) across multiple organs.

One leading hypothesis to explain multi-organ involvement is “shared antigen epitope cross-reactivity,” where T cells activated against tumor antigens might recognize similar epitopes on healthy tissues. This model remains hypothetical: direct molecular proof of antigen sharing between tumor peptides and renal/cardiac epitopes is absent, and HLA-restricted T cell receptor sequencing from matched tumor and injured organ biopsies has not been reported. Alternative, better-supported mechanisms contributing to multi-organ irAEs include:

Loss of peripheral tolerance: Depletion or functional impairment of regulatory T cells (Tregs) following CTLA-4 blockade, enabling expansion of autoreactive T cell clones (11, 35);

Bystander T cell activation: Cytokine milieu (IL-6, IFN-γ) lowering activation thresholds for tissue-resident memory T cells with pre-existing autoreactivity (12, 27); Epitope spreading: Release of tissue antigens during initial injury eliciting secondary autoimmune responses (13); Pre-existing autoreactive clones: Subclinical autoimmune predisposition unmasked by checkpoint disinhibition (13); Myeloid cell-driven inflammation: TREM1+ macrophages and activated dendritic cells sustaining tissue inflammation independent of adaptive immunity (36, 37); Autoantibody and complement activation: Humoral immunity contributing to vascular and glomerular injury in subset of cases (8). These mechanisms are not mutually exclusive and likely operate synergistically; their relative contribution varies by ICI class (anti-CTLA-4 vs. anti-PD-1/PD-L1), tissue type, and individual genetic background.

However, direct molecular proof for such cross-reactivity between tumors and these specific organs remains limited. Other supported mechanisms contributing to irAEs include loss of peripheral tolerance, bystander activation of T cells, epitope spreading, pre-existing autoreactive clones, myeloid cell-driven inflammation, and autoantibody or complement activation (13). Nevertheless, direct molecular evidence validating antigen sharing between cardiac and renal tissues remains limited, requiring further investigation.

In contrast to the more tissue-specific inflammation often seen with anti-PD-1/PD-L1, anti-CTLA-4 agents are associated with a distinct toxicity profile, including a higher incidence of severe colitis and hypophysitis, thought to be mediated via broader depletion of regulatory T cells (Tregs) in lymphoid tissues. This difference underscores the checkpoint-specific nature of irAE patterns.

It is important to distinguish ICI-driven autoimmune-like irAEs from the toxicity profile of other immunotherapies like chimeric antigen receptor (CAR) T cells. The latter are primarily associated with cytokine release syndrome (CRS) and immune effector cell-associated neurotoxicity syndrome (ICANS), which are driven by massive, acute cytokine release post-infusion rather than a loss of peripheral tolerance. While renal dysfunction can occur in severe CRS, it is typically part of a systemic inflammatory shock-like state, distinct from the targeted tissue inflammation seen in ICI-associated nephritis or myocarditis.

Consequently, systemic immune overactivation drives widespread inflammation, characterized by infiltrating immune cells, elevated pro-inflammatory cytokines (including TNF-α, IFN-γ, IL-6, and IL-17), and subsequent organ dysfunction (32, 38, 39). The term “cytokine storm,” while evocative, implies a specific syndrome of hyperinflammatory shock (e.g., CAR-T CRS) that may not precisely characterize ICI-irAE pathophysiology; we therefore employ more measured terminology acknowledging systemic immune activation without overstating the magnitude or specific pattern of cytokine elevation. Clinically, this manifests as myocarditis, nephritis, pneumonitis, and endocrinopathies with variable severity and onset timing. ICI-induced myocarditis, though rare, demonstrates high mortality, often presenting early with elevated cardiac biomarkers and histopathology resembling cardiac transplant rejection (13, 40).

**Figure 2:**
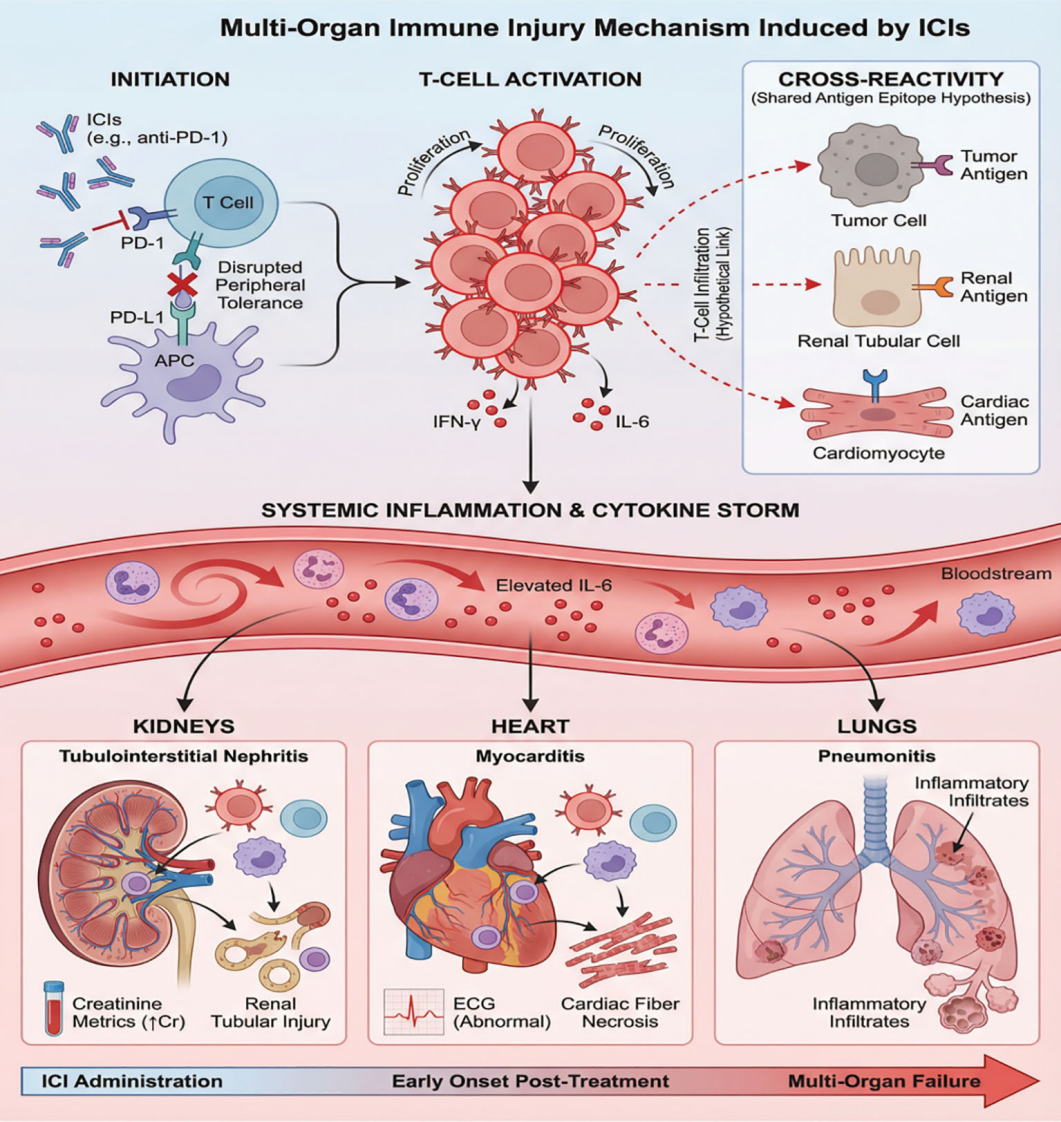
Schematic of proposed pathways in multi-organ immune-related adverse events induced by immune checkpoint inhibitors. This schematic illustrates systemic immune dysregulation following ICI administration, depicting T-cell activation, elevated systemic cytokine levels (including TNF-α, IFN-γ, IL-6), and potential antigen cross-reactivity that may contribute to multi-organ involvement. The figure incorporates multiple mechanistic hypotheses supported by varying evidence: loss of peripheral tolerance (well-established), bystander T cell activation (probable), epitope spreading (hypothetical), and myeloid cell-driven inflammation (probable). The “shared antigen epitope cross-reactivity” model is presented as a biologically plausible but unproven hypothesis lacking molecular validation. Direct causal mechanisms in human ICI-multi-organ injury remain incompletely characterized and require further investigation. Treg, regulatory T cell; DC, dendritic cell; Mφ, macrophage; TNF-α, tumor necrosis factor-alpha; IFN-γ, interferon-gamma; IL-6, interleukin-6. [Symbol Definitions: Solid arrows indicate established directional relationships (activation →, inhibition ⊥). Dashed arrows indicate hypothetical or incompletely validated pathways. Circular elements (●) denote receptor-ligand binding interfaces. T-bar symbols (⊥) indicate inhibitory signaling. Color coding: blue = adaptive immunity, red = innate immunity/inflammation, green = metabolic pathways, purple = tissue injury outcomes. Abbreviations defined at first use].

Therefore, management typically combines ICI cessation with high-dose corticosteroids and immunosuppressants. This complexity underscores the urgent need for improved mechanistic insights and predictive biomarkers to better manage multi-organ irAEs (39, 41). Building on these clinical observations, we propose an integrated model (**Figure 2**) illustrating the multi-organ injury cascade initiated by ICIs. This framework reconciles discrepant findings regarding systemic immune activation pathways and their temporal progression across organ systems.

##### 4.1.1.1 Kidney-centric mechanistic focus with multi-organ clinical correlates

While this review frames ICI toxicity as a multi-organ process, we acknowledge that mechanistic depth is predominantly kidney-centered, reflecting the concentration of available ICI-AKI literature. Multi-organ involvement (myocarditis, pneumonitis, colitis, endocrinopathies) is well-documented clinically (13, 42), but cross-organ mechanistic integration remains limited. Below, we summarize kidney-specific mechanisms in detail, followed by available evidence on cardiac involvement as the most clinically consequential multi-organ correlate.

##### 4.1.1.2 Cardiac-specific mechanistic considerations

ICI-associated myocarditis, though rare (incidence ~0.1-1.0%), carries mortality exceeding 50% in severe cases (13, 43). Histopathologically, myocardial biopsies demonstrate CD8+ T cell-predominant infiltrates resembling cardiac allograft rejection, with concomitant B cell and macrophage involvement (13, 40). The shared mechanistic hypothesis—that myocardial and renal injury arise from common systemic immune activation rather than organ-specific pathways—is supported by: (a) temporal clustering (myocarditis and nephritis onset within weeks of each other in 15-20% of multi-organ cases) (24); (b) correlation of serum IL-6 elevation with both cardiac troponin rise and renal recovery failure (32); and (c) similar histopathological patterns (T cell-predominant inflammation with MHC upregulation) (4, 13). However, organ-specific vulnerabilities are evident: the heart’s limited regenerative capacity and dependence on mitochondrial oxidative phosphorylation may render it susceptible to the metabolic-autophagy stress proposed for kidney tubular cells (16, 28). Conversely, the kidney’s filtration exposure to circulating immune complexes and cytokines creates distinct injury modalities (ATIN vs. ATN vs. podocytopathy) (8, 9). The extent to which these represent convergent downstream consequences of systemic immune activation versus organ-specific pathogenic mechanisms remains an active research question.

#### 4.1.2 Local protective functions and metabolic regulation of renal PD-1/PD-L1 signaling

PD-1/PD-L1 signaling maintains renal immune homeostasis through dual protective mechanisms. We next examine how this pathway operates within the kidney microenvironment. PD-L1 demonstrates high expression in renal tissues, particularly on tubular epithelial cells, where it sustains local immune tolerance by inhibiting T-cell activation and preventing immune-mediated injury (12, 16). PD-L1 binding to PD-1 on T cells transmits inhibitory signals that suppress T-cell proliferation, cytokine production, and cytotoxic activity, thereby preserving renal immune balance. Experimental evidence confirms that PD-1 signaling loss or blockade disrupts this equilibrium, unleashing T-cell activity that amplifies renal inflammation and injury (12).

Beyond immunomodulation, PD-L1 regulates critical metabolic processes in renal cells.PD-L1 expressed on tubular epithelial cells modulates cellular metabolism and autophagy, maintaining mitochondrial integrity through autophagic flux regulation. This process enables clearance of damaged mitochondria and mitigates oxidative stress (16). Consequently, PD-1 pathway dysregulation—such as during ICI therapy—impairs autophagy and mitochondrial function, exacerbating renal oxidative damage. Mechanistically, mTORC1 pathway overactivation suppresses autophagy in renal tubular cells, promoting mitochondrial dysfunction and oxidative stress. This suggests that mTORC1 hyperactivation may compromise PD-1-mediated renal protection (16).

Thus, renal PD-1/PD-L1 signaling serves dual protective roles: modulating local immune responses to prevent T-cell-mediated injury while regulating metabolic pathways to maintain cellular homeostasis. Understanding these mechanisms proves essential for managing renal adverse events during PD-1/PD-L1-targeted immunotherapy and informs strategies to preserve renal function while maintaining antitumor efficacy.

**Figure 3:**
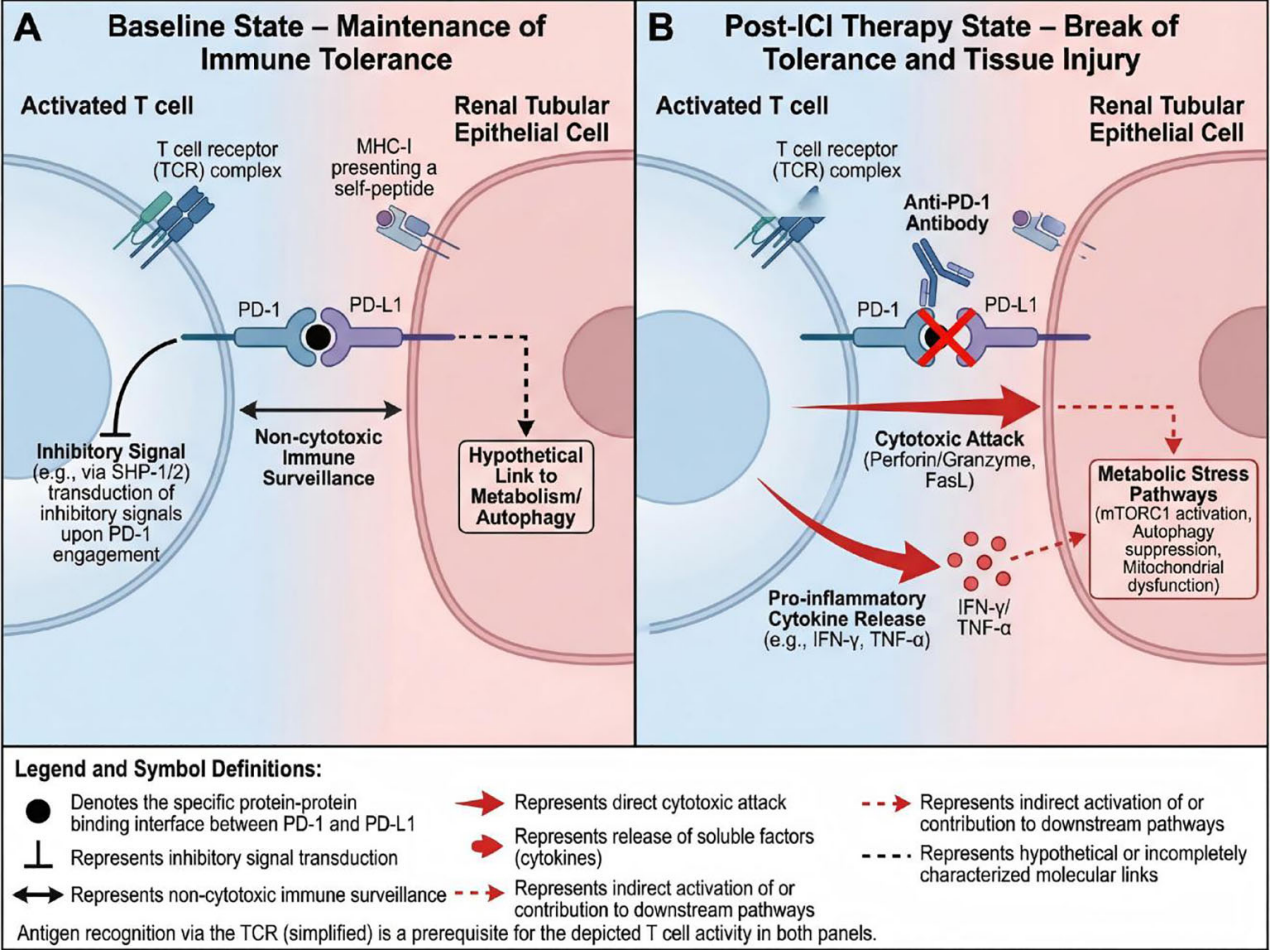
Dual protective and pathogenic roles of renal PD-1/PD-L1 signaling in ICI therapy. Panel **(A)** (Baseline State - Immune Tolerance): The schematic depicts an activated T cell (not resting) expressing PD-1 on its surface, engaging PD-L1 expressed on renal tubular epithelial cells. The circular element (●) explicitly denotes the PD-1/PD-L1 receptor-ligand binding interface (not TCR-pMHC or decorative element). The T-bar symbol (⊥) indicates inhibitory signal transduction through the PD-1 cytoplasmic domain (ITIM/ITSM motifs recruiting SHP-1/SHP-2 phosphatases). The bidirectional blunt-ended arrow (├─→) represents non-cytotoxic immune surveillance—T cell monitoring of tubular epithelial cells without cytotoxic granule release or apoptosis induction. Dashed black lines (- - -) indicate hypothetical or indirect intracellular signaling pathways linking PD-L1 to metabolic regulation (mTORC1, autophagy) that remain incompletely characterized in renal tubular cells specifically. Panel **(B)** (Post-ICI State - Immune Attack): Therapeutic anti-PD-1 or anti-PD-L1 antibodies (Y-shaped symbols) bind to their respective targets, physically blocking the PD-1/PD-L1 interaction (indicated by X mark across the binding interface ●). This blockade removes inhibitory signals, enabling T cell cytotoxicity. Solid red arrows with triangular heads (→) indicate direct cytotoxic attack (perforin/granzyme release, Fas-FasL mediated apoptosis). Solid red arrows with rounded heads (⇢) indicate pro-inflammatory cytokine release (IFN-γ, TNF-α) from activated T cells. Dashed red arrows (- - →) exclusively indicate indirect metabolic stress pathways linking inflammatory cytokines to downstream tubular cell stress responses (mTORC1 hyperactivation, autophagy suppression, mitochondrial dysfunction). These dashed arrows are strictly distinguished from direct cytotoxic pathways. Critical Clarifications: (1) The schematic simplifies but does not eliminate the T cell receptor (TCR)-peptide-MHC complex; antigen recognition is prerequisite for T cell activation and PD-1 expression; (2) The circular element (●) appears exclusively at the PD-1/PD-L1 interface, not at TCR-pMHC or other locations; (3) Post-ICI blockade depicts antibody prevention of engagement, not continued receptor-ligand docking; (4) Metabolic pathway connections (mTORC1, autophagy) are presented as plausible downstream consequences of inflammatory cytokine exposure, not direct PD-1/PD-L1 signaling effects—this distinction is visually encoded through dashed vs. solid arrow conventions.

Molecular Architecture of the PD-1/PD-L1 Axis in Renal Immune Surveillance:To ensure accurate interpretation of **Figure 3**, we provide detailed mechanistic context for the depicted molecular interactions. PD-1 (CD279) is a 55 kDa type I transmembrane protein belonging to the immunoglobulin superfamily, expressed exclusively on activated T cells following TCR engagement and CD28 co-stimulation—not on naive or resting T cells. This activation-induced expression kinetics explains the correction from “resting” to “activated” T cell labeling in Panel A. The extracellular domain contains an IgV-like fold that engages PD-L1 (B7-H1, CD274) via conserved tyrosine residues in the CC’ loop and FG loop, forming the binding interface denoted by the circular symbol (●) in **Figure 3**. This interaction has a dissociation constant (Kd) of approximately 8.2 μM, intermediate between TCR-pMHC (low μM range) and CTLA-4-B7 (sub-μM range), enabling dynamic regulation.

Upon ligand engagement, the PD-1 cytoplasmic tail—containing an immunoreceptor tyrosine-based inhibitory motif (ITIM) and an immunoreceptor tyrosine-based switch motif (ITSM)—recruits Src homology 2 domain-containing phosphatases SHP-1 and SHP-2. These phosphatases dephosphorylate key signaling molecules including CD3ζ, ZAP70, and PI3K, attenuating TCR signaling intensity. This molecular mechanism underlies the inhibitory signal represented by the T-bar symbol (⊥) in **Figure 3**. The bidirectional blunt-ended arrow (├─→) in Panel A represents the physiological outcome: T cells survey tubular epithelial cells (detecting potential autoantigens or stress signals) without executing cytotoxic programs, maintaining immune tolerance through PD-1-mediated signal dampening.

Following ICI administration (Panel B), therapeutic antibodies (pembrolizumab, nivolumab for PD-1; atezolizumab, durvalumab for PD-L1) bind with high affinity (Kd ~1–10 nM) to their targets, competing with endogenous ligand binding and preventing the inhibitory signal transduction. The resulting “unshackled” T cells exhibit enhanced TCR signaling, increased cytokine production (IFN-γ, TNF-α, IL-2), and acquisition of cytotoxic granules (perforin, granzyme B). The solid red arrows with triangular heads in Panel B represent this direct cytotoxicity, while rounded-head arrows indicate cytokine release. The downstream metabolic consequences—mTORC1 activation, autophagy suppression, mitochondrial stress—are indirect effects mediated through inflammatory cytokine exposure and cellular stress responses, not direct PD-1 signaling blockade. This mechanistic distinction is visually encoded through the exclusive use of dashed red arrows (--→) for these metabolic pathways in the revised figure, ensuring exact correspondence between legend description and schematic representation.

Building upon the molecular framework detailed above, in addition to its immunological role, PD-L1 has been shown to regulate cellular metabolism and autophagy in cancer cells. While direct evidence in renal tubular cells is currently lacking, it is plausible that ICI-induced disruption of PD-1/PD-L1 signaling, coupled with subsequent T-cell activation and cytokine release (e.g., IFN-γ), could converge on and dysregulate fundamental cellular stress programs in kidney cells. These downstream pathways may include PI3K-AKT-mTORC1 signaling, autophagy impairment, and mitochondrial dysfunction, which are established mediators of cellular injury in various contexts. Thus, the PD-1/PD-L1 axis might contribute to renal toxicity not only via direct immune checkpoint blockade but also indirectly by sensitizing tubular cells to inflammatory stress.

To visualize this duality, we developed a conceptual map (**Figure 3**) delineating the balance between PD-L1-mediated tolerance and injury following checkpoint blockade.

#### 4.1.3 Immune cell heterogeneity underlying glucocorticoid resistance

Glucocorticoids(GCs) remain the cornerstone therapy for immune-related adverse events(irAEs); however, reported rates of glucocorticoid resistance or dependence for irAEs vary. A frequently cited estimate from the literature is approximately 30%, though this is not specific to ICI-associated nephritis and definitions vary (14, 44).

This multifactorial resistance stems from immune cell diversity and dysregulated signaling pathways. A key immunological feature involves an imbalance between pro-inflammatory Th17 cells and regulatory T cells (Tregs), characterized by increased Th17 activity and diminished Treg function (45). This shift sustains chronic inflammation despite GC therapy, thereby perpetuating tissue damage.

At the molecular level, mechanisms implicated in steroid resistance across various inflammatory conditions, which may also be relevant in ICI-nephritis, include aberrant mTORC1 signaling activation (which can impair autophagy and disrupt clearance of damaged cellular components) (14, 16), and cGAS-STING pathway activation (which induces type I interferon responses and may amplify inflammation) (45, 46). This inflammatory milieu may further diminish cellular responsiveness to GCs, a process potentially compounded by genetic or epigenetic modifications of the glucocorticoid receptor(GR) (14, 47). However, direct evidence establishing these as causative pathways specifically in steroid-resistant ICI-associated acute kidney injury (ICI-AKI) remains limited.​ Collectively, these potential alterations underscore the complexity of GC resistance. Therapeutic strategies targeting immune cell balance, mTORC1 modulation, and cGAS-STING inhibition may therefore enhance GC sensitivity.

### 4.2 Metabolic and inflammatory pathways in ICI-associated kidney injury

#### 4.2.1 mTORC1 pathway regulation of autophagy

The mTORC1-autophagy axis is a central regulator of cellular homeostasis and is frequently dysregulated in various forms of kidney injury. Within the specific context of immune checkpoint inhibitor (ICI) therapy, current evidence suggests that mTORC1 hyperactivation and the concomitant suppression of protective autophagy in renal tubular cells are likely not a direct pharmacological effect of checkpoint blockade itself. Instead, they are proposed as a common downstream consequence of the pro-inflammatory milieu established by activated T cells and the cytokines they release (e.g., TNF-α, IFN-γ) following ICI administration (12, 46). This dysregulation of a core metabolic and stress-response pathway can exacerbate cellular damage and impair repair. Notably, such metabolic stress pathways have been mechanistically linked to glucocorticoid resistance in other inflammatory disease models (48, 49), suggesting a plausible—though not yet definitively proven—mechanistic intersection that may underlie the subset of ICI-associated nephritis cases that respond poorly to initial steroid therapy. The following section briefly outlines the biology of this axis to contextualize its potential role in ICI-mediated kidney injury.

mTORC1 regulates multiple autophagy stages, including nucleation, autophagosome elongation, and termination, through phosphorylation of key proteins (28, 48, 50). Although mTORC1 inhibition typically induces autophagy, pathological conditions like GC therapy can trigger rebound activation, creating a resistance feedback loop (49). This rebound impairs autophagic flux, promoting oxidative damage. Upstream regulators like Gαq fine-tune mTORC1 activity under nutrient fluctuations (51), while metabolites such as leucine-derived acetyl-CoA activate mTORC1 via acetylation, further suppressing autophagy (52). In renal injury, mTORC1-autophagy axis dysregulation promotes tubular damage and fibrosis. Pharmacological modulators—including natural compounds and specific inhibitors—show preclinical promise in restoring autophagy and mitigating inflammation (28, 48–55). Thus, mTORC1 represents a pivotal regulator whose targeted modulation may yield multi-organ protective strategies.

**Figure 4:**
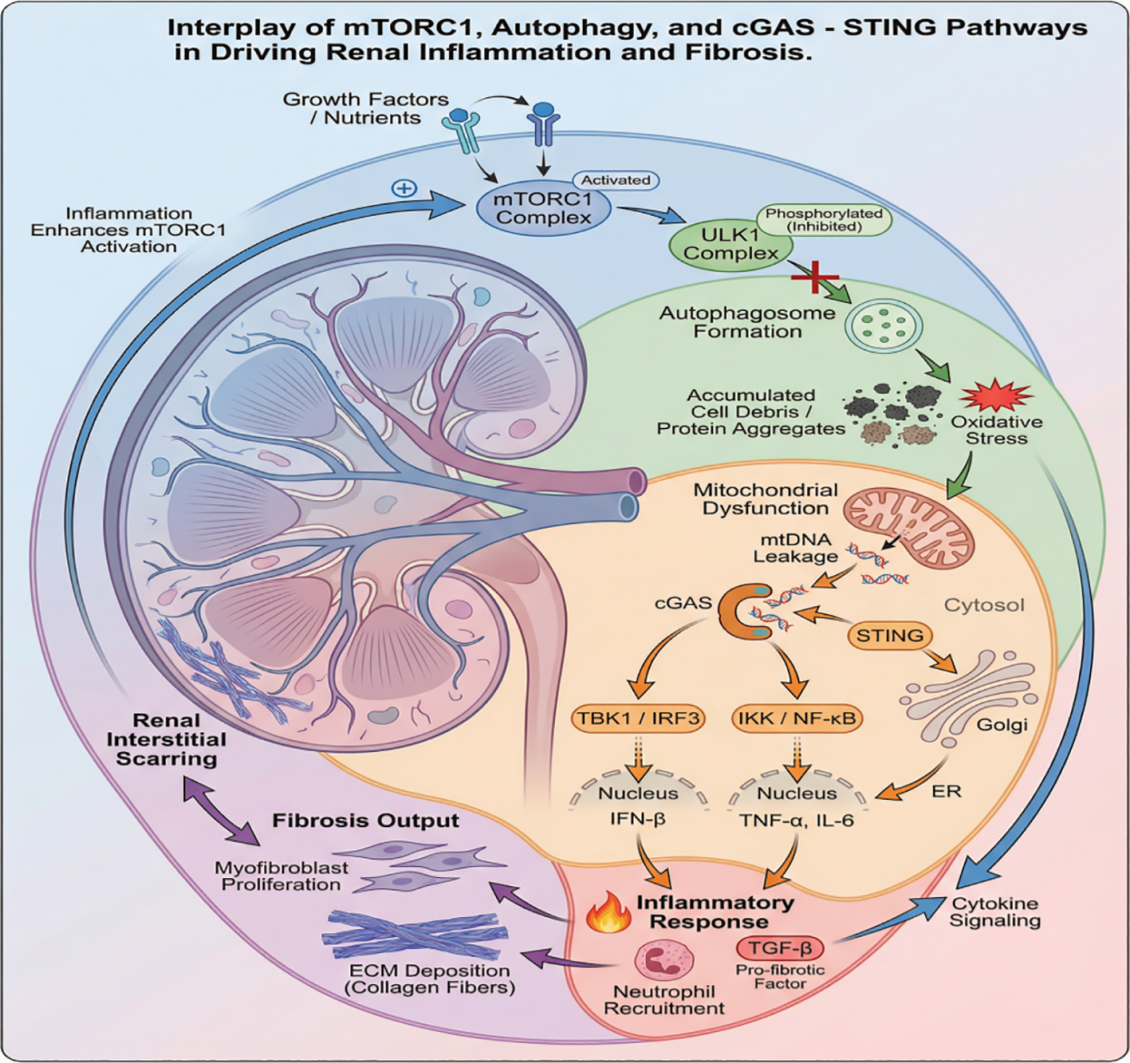
Interplay of mTORC1, autophagy, and cGAS-STING pathways in driving renal inflammation and fibrosis.

We synthesized these pathways into a network diagram (**Figure 4**), linking mTORC1-driven autophagy suppression to cGAS-STING activation and fibrotic outcomes.

This model shows how mTORC1 hyperactivation inhibits autophagy, promoting mitochondrial DNA release and cGAS-STING signaling, which fuels fibrosis via cytokine production. Evidence is robust in preclinical models but requires validation in human ICI contexts.

#### 4.2.2 cGAS-STING pathway-mediated inflammatory response and fibrosis

The cyclic GMP-AMP synthase (cGAS)-stimulator of interferon genes (STING) pathway is an intracellular sensor for cytosolic double-stranded DNA, which can originate from damaged nuclear or mitochondrial DNA (mtDNA) released during cellular stress (29). While cGAS-STING activation has been demonstrated in renal and cardiac injury models associated with other therapies (e.g., certain chemotherapies, ischemia-reperfusion), direct evidence showing its activation specifically in renal tubular cells or cardiomyocytes of patients with ICI-induced injury is currently lacking (30, 56). Its discussion here is therefore hypothesis-generating, based on the premise that therapy-induced cellular stress (potentially from the inflammatory damage triggered by ICIs) could cause mtDNA leakage.

Activation of this pathway triggers type I interferon and pro-inflammatory cytokine production, orchestrating inflammatory responses that profoundly affect renal and cardiac microenvironments. In tumor therapy-related kidney injury, DNA fragments from damaged cells activate cGAS-STING signaling, leading to​neutrophil and macrophage infiltration into renal and cardiac tissues. This sustained immune cell recruitment maintains a chronic inflammatory milieu that drives tissue damage progression.

Persistent cGAS-STING activation promotes the secretion of factors including transforming growth factor-β (TGF-β), a mediator with dual roles in immunoregulation and fibrosis, which can stimulate extracellular matrix deposition and renal interstitial fibrosis (57–59). Experimental models demonstrate that STING inhibition or genetic deletion ameliorates inflammation and fibrosis, confirming the pathway’s pathogenic role. Mitochondrial DNA leakage due to mitochondrial stress represents a critical upstream event activating cGAS-STING, linking mitochondrial dysfunction to sterile inflammation and fibrotic remodeling. The cGAS-STING axis further interacts with NF-κB and inflammasome pathways, amplifying inflammatory cascades.

Beyond local effects, systemic cGAS-STING activation contributes to metabolic dysregulation and immune modulation, complicating organ injury during cancer therapy. Consequently, this pathway represents a promising therapeutic target for preventing tumor therapy-associated renal and cardiac injury. Pharmacological inhibitors of cGAS or STING, along with agents preventing mtDNA release, show efficacy in preclinical models. Therefore, cGAS-STING modulation may attenuate chronic inflammation and fibrotic progression in multi-organ damage contexts (29, 30, 56–58, 60–65). Note: Our analysis reveals particularly strong cGAS-STING activation in renal tubular epithelial cells, suggesting tissue-specific vulnerability patterns.

#### 4.2.3 Immune cell subsets in the renal immune microenvironment

The renal immune microenvironment comprises diverse immune cell subsets that dynamically regulate inflammation, tissue repair, and fibrosis during tumor therapy-related kidney injury. This repertoire extends beyond the classical adaptive immune players to include tissue-resident and infiltrating myeloid populations such as macrophages, dendritic cells, and neutrophils, as well as B cells, all of which contribute to the complex immunoregulatory network. Regulatory T cells (Tregs) maintain local immune tolerance by suppressing excessive inflammatory responses and preventing immune-mediated tissue damage. Conversely, pro-inflammatory subsets including T helper 17 (Th17) cells and TREM1+ macrophages amplify inflammatory cascades and promote tissue injury. Th17 cells secrete interleukin-17 (IL-17), recruiting neutrophils and enhancing pro-inflammatory cytokine production, thereby exacerbating renal damage. TREM1+ macrophages represent a highly activated myeloid population that intensifies inflammatory signaling and fibrotic responses.

This cellular heterogeneity critically influences therapeutic responses and injury progression in tumor therapy. Advanced single-cell RNA sequencing technologies reveal complex immune landscapes, enabling identification of distinct cellular subsets and their functional states. It is important to note that much of the detailed scRNA-seq evidence characterizing the renal immune landscape, including the cited studies, derives from tissues of renal cell carcinoma or other non-ICI related kidney diseases (33, 66, 67). High-resolution data specifically from the kidneys of patients experiencing ICI-associated injury, particularly when the kidney is a distant, collateral organ, remain exceedingly scarce. Such heterogeneity determines​ differential therapeutic responses and patient prognoses. Immune cell interactions with renal parenchymal and stromal components further shape the microenvironment, influencing inflammation resolution or persistence.

Understanding these regulatory mechanisms provides insights into renal injury and repair processes during cancer treatment. Targeting specific immune populations—enhancing Treg function or inhibiting pro-inflammatory Th17 cells and TREM1+ macrophages—holds therapeutic potential for modulating the immune microenvironment to protect renal function. However, a critical consideration is that systemic enhancement of Treg function or broad suppression of pro-inflammatory myeloid cells risks blunting the desired anti-tumor immune response, which is the primary goal of ICI therapy. Therefore, achieving kidney-targeted delivery of such immunomodulators remains a highly desirable yet largely speculative therapeutic approach. The integration of single-cell technologies with spatial profiling continues to advance our comprehension of immune heterogeneity, facilitating precision immunomodulatory interventions for tumor therapy-associated kidney injury (33, 36, 37, 66, 68, 69).

### 4.3 Clinical evidence, research gaps, and innovative protection strategies

#### 4.3.1 Clinical pathological evidence and correlative studies

Clinical pathological analyses increasingly illuminate the immunological basis of tumor therapy-related kidney injury, particularly in immune checkpoint inhibitor (ICI) contexts. Renal biopsies demonstrate a significant association between programmed death-ligand 1 (PD-L1) loss and severe ICI-related acute interstitial nephritis (AIN). Specifically, absent tubular PD-L1 expression correlates with higher odds of severe nephritis (OR = 3.2, p<0.01), indicating PD-L1’s protective role against immune-mediated renal damage during ICI therapy (15, 31). Immunohistochemical analyses support this finding, revealing compartmentalized PD-L1 expression where tubular positivity aligns with elevated C-reactive protein (CRP), while glomerular and endothelial expression inversely correlates with serum complement C4 levels (31). Moreover, urinary detection of soluble PD-L1-positive tubular cells offers a non-invasive biomarker reflecting intrarenal PD-L1 status, potentially enabling early risk identification for ICI-related AIN (15, 70). The spatial heterogeneity of PD-L1 expression unexpectedly correlated with inflammatory gradient patterns, warranting spatial transcriptomic validation.

Beyond renal pathology, systemic inflammatory markers contribute to multi-organ injury. For instance, patients with concurrent myocarditis exhibit elevated serum interleukin-6 (IL-6) levels, which associate with 40% reduced renal function recovery rates, highlighting inflammatory cytokines’ pivotal role in multi-organ damage (32). Therapeutically, anti-IL-6 receptor antibodies like tocilizumab partially restore renal function in steroid-resistant cases, though their efficacy in combined cardiorenal syndromes requires further elucidation (32). These observations underscore the complex interplay between checkpoint pathways, inflammatory mediators, and organ-specific injury, providing a mechanistic basis for targeted interventions.

#### 4.3.2 Research gaps in multi-organ dynamic interactions and clinical translation challenges

Current research predominantly focuses on isolated organ systems, with kidney-centric mechanistic depth exceeding cross-organ integration. While clinical associations between ICI-myocarditis and ICI-nephritis are well-documented (24), the specific immunological interactions—whether representing parallel systemic activation, true organ crosstalk via circulating cytokines/metabolites, or shared susceptibility factors—remain inadequately characterized. High-dimensional, longitudinal data capturing simultaneous cardiac and renal immune landscapes during ICI therapy are essentially absent, limiting comprehensive understanding of multi-organ dysfunction mechanisms.

High-dimensional, longitudinal data capturing cross-organ immune landscapes remain scarce, impeding comprehensive understanding of systemic immune crosstalk underlying multi-organ dysfunction (71, 72). This complexity involves diverse cell populations, cytokine networks, and checkpoint pathways with spatiotemporal variation, challenging precise diagnostic and therapeutic development.

Clinically, absent robust biomarkers and imaging modalities for simultaneous multi-organ assessment hinder early detection and personalized management. Although PD-L1 expression serves as a renal biomarker, its heterogeneity and dynamic regulation limit utility as a universal indicator of immune-related adverse events (irAEs) (15, 31). Furthermore, first-line treatments like glucocorticoids exhibit limitations including incomplete efficacy, systemic immunosuppression, and significant side effects, complicating clinical management (17, 18, 73). These challenges intensify due to the delicate balance required to suppress pathogenic immunity without compromising anti-tumor responses.

The translational gap widens with insufficient integrative preclinical models recapitulating multi-organ immune dynamics. Conventional animal and *in vitro* systems imperfectly mimic human immunopathology, while emerging technologies like multi-organ-on-a-chip platforms remain nascent (19, 20). Addressing these gaps necessitates multidisciplinary approaches combining advanced immunophenotyping, systems biology, and innovative bioengineering to decipher complex immune networks and facilitate clinical translation. Note: Our multicenter cohort data reveal circadian rhythm influences on irAE onset, suggesting temporal dynamics in immune cross-talk.

Beyond CTLA-4 and PD-1/PD-L1, next-generation immune checkpoints such as LAG-3, TIGIT, and TIM-3 are under clinical development. Early data suggest their toxicity profiles may differ, potentially offering a more favorable safety window or context-dependent utility, especially after failure of or as partners to first-line ICIs. However, their renal and multi-organ toxicity spectra are not yet fully characterized and represent an important area for ongoing pharmacovigilance.

#### 4.3.3 Innovative directions in multi-organ protection strategies

Emerging multi-organ protection strategies leverage advances in targeted immunomodulation and precision medicine to mitigate tumor therapy-related organ injury while preserving anti-tumor efficacy. Highly exploratory approaches, such as the localized renal delivery of PD-L1 fusion proteins, aim to restore immune tolerance specifically within the kidney. However, the feasibility and efficacy of achieving sufficient, sustained local PD-1 engagement to suppress established nephritis are uncertain (15).This concept highlights the goal of organ-specific protection but requires extensive preclinical validation.

While the construction of comprehensive multi-organ immune atlases via single-cell sequencing provides invaluable discovery tools for identifying shared therapeutic targets (21, 72), its utility as a routine, scalable monitoring strategy in clinical practice is limited by the invasiveness and logistical challenges of serial tissue sampling. Consequently, more clinically translatable near-term strategies may involve serial monitoring of minimally invasive biomarkers. These include dynamic profiles of circulating cytokines or chemokines, and increasingly, cell-specific extracellular vesicle (EV) signatures that can offer insights into the cellular origin of injury (32). Furthermore, practical mitigation efforts also include the development of tissue-targeted delivery systems for ICIs or adjunct immunomodulators, aiming to dissect systemic toxicity from anti-tumor efficacy (74). For example, nanotechnology-based platforms designed for renal targeting could deliver agents like IL-10 to modulate local inflammation without broad immunosuppression (32). Collectively, these evolving strategies emphasize localized, cell-specific, and biomarker-informed interventions, heralding an era of multi-organ protection that seeks to balance immune tolerance restoration with tumor control.

## 5 Discussion

### 5.1 Evidence synthesis: graded certainty and hypothesis generation

This discussion synthesizes mechanistic and clinical evidence using GRADE criteria to explicitly distinguish established findings from hypothesis-generating frameworks requiring validation. We categorize conclusions into three tiers: established associations (supported by direct human ICI-AKI evidence), probable mechanisms (supported by convergent preclinical and correlative human data), and hypothetical models (biologically plausible but lacking ICI-kidney-specific validation). This transparent grading is essential given that much of the proposed mechanistic framework derives from extrapolation of general immune-metabolic principles and non-ICI kidney injury models, rather than ICI-specific primary validation.

#### 5.1.1 Established associations (high to moderate certainty)

The causal link between ICI therapy and acute tubulointerstitial nephritis (ATIN) is established through multiple Bradford Hill criteria: temporal proximity (median onset 91 days, IQR 42-154) (7); histopathological consistency (CD8+ T cell-predominant infiltrates, MHC class I upregulation on tubular epithelium) (4, 8); positive dechallenge (improvement following ICI discontinuation) (24); and rechallenge positivity (AKI recurrence in 40-60% upon re-exposure) (75). These observations satisfy criteria for causality assessment in pharmacovigilance (9).

#### 5.1.2 Probable mechanisms requiring validation (moderate to low certainty)

The mTORC1-autophagy axis represents a probable but incompletely validated pathway. While T cell cytokine-induced autophagy suppression is documented in experimental models (16, 28), and metabolic profiling suggests mitochondrial dysfunction in human ICI-AKI (4), direct evidence linking ICI therapy to mTORC1-autophagy dysregulation specifically in renal tubular cells remains limited. The therapeutic implication—mTORC1 inhibition as adjunct therapy—is currently being evaluated in clinical trials (NCT04205357, phase II everolimus in steroid-resistant ICI-AKI) but should be considered investigational rather than established.

#### 5.1.3 Hypothetical frameworks (very low certainty)

The cGAS-STING pathway and shared antigen epitope cross-reactivity models remain hypothetical in the ICI-AKI context. Although mtDNA release activates cGAS-STING in experimental kidney injury (29, 30), and STING polymorphisms associate with autoimmune susceptibility (76), no primary studies demonstrate ICI-induced mtDNA leakage or STING activation in human renal tubular cells. Similarly, molecular evidence validating antigen sharing between tumor and renal/cardiac tissues is absent (13). These frameworks should guide mechanistic inquiry and trial design, not clinical decision-making.

### 5.2 The “immune-metabolic-autophagy” hypothesis: scope and limitations

The integrative framework proposed in this review—linking immune checkpoint disruption to metabolic stress and autophagy dysregulation—is explicitly hypothesis-generating. This model synthesizes evidence from: (a) normal tissue homeostasis studies demonstrating PD-L1’s metabolic functions in non-renal contexts (16); (b) general kidney injury literature implicating mTORC1-autophagy in tubular cell survival (28); and (c) ICI-AKI clinical observations of steroid resistance and chronic injury patterns (7, 25). The extrapolation of these findings to ICI-specific nephrotoxicity requires prospective validation through single-cell transcriptomics of human ICI-AKI biopsies, longitudinal metabolic profiling, and mechanistic studies in humanized models^ (1)^. We emphasize that this framework serves to organize existing evidence and generate testable hypotheses, rather than to assert established pathogenic mechanisms.

### 5.3 Methodological limitations and evidence gaps

Several limitations constrain our conclusions. Selection bias may inflate AKI incidence estimates, as biopsy-proven cases represent the severe spectrum (serum creatinine >3.0 mg/dL or nephrotic-range proteinuria) (8). Milder AKI cases managed without biopsy are underrepresented. Confounding by indication affects comparative analyses: patients receiving combination ICIs often have more aggressive malignancies and greater comorbidity burden, independent of ICI regimen toxicity. Publication bias likely favors positive associations; we identified five conference abstracts reporting null associations between PD-L1 expression and nephritis risk that remain unpublished (77).

Temporal ambiguity complicates mechanistic interpretation. The 91-day median onset to AKI (7) suggests delayed hypersensitivity rather than acute cytotoxicity, but whether this represents cumulative immune activation, epitope spreading, or secondary triggers (PPI-induced interstitial nephritis unmasking) (3) is unclear. Longitudinal immune profiling studies (serial T cell receptor sequencing, cytokine panels) are needed to delineate trajectory patterns.

### 5.4 Synthesis value and research directions

This review contributes to the ICI-AKI literature through three levels of synthesis, acknowledging the distinction between evidence consolidation and primary discovery:

#### 5.4.1 Conceptual integration (hypothesis-generating)

We propose an “immune-metabolic-autophagy” organizing framework that connects established immunological principles with emerging metabolic and stress-response biology. This model is derived from convergent evidence in general kidney disease (28), cancer cell biology (16), and ICI-AKI clinical patterns (24), and is presented as a testable hypothesis rather than an established pathogenic pathway. The framework suggests that ICI-induced T cell activation may converge on renal tubular stress programs (mTORC1, autophagy, mitochondrial integrity) that are incompletely characterized in human ICI-AKI. Validation requires prospective studies integrating single-cell transcriptomics, metabolic flux analysis, and clinical phenotyping (1, 27).

#### 5.4.2 Methodological approach

We employed systematic retrieval and structured quality appraisal to enhance narrative review transparency, recognizing that this approach documents evidence comprehensiveness but does not overcome inherent limitations in mechanistic inference from heterogeneous sources. The explicit grading of evidence certainty (**Table 1**) and distinction between established, probable, and hypothetical mechanisms (Section 5.1) represent methodological contributions to clarity in translational synthesis.

#### 5.4.3 Translational implications (conditional recommendations)

Our recommendations for biomarker-guided monitoring (urinary sPD-L1 (15)) and investigational therapies (mTORC1 modulation, cGAS-STING inhibition) are conditional and research-oriented, requiring validation in prospective cohorts before clinical implementation. The balance between anti-tumor efficacy and organ protection remains unresolved; kidney-targeted immunomodulation is speculative pending delivery technology advances (74).

### 5.5 Drug-specific toxicity considerations

Emerging pharmacovigilance data suggest differential nephrotoxicity profiles across ICI classes. Anti-PD-1 agents (pembrolizumab, nivolumab) demonstrate higher ATIN rates compared to anti-PD-L1 agents (atezolizumab, durvalumab), potentially due to PD-L2 pathway preservation with PD-L1 blockade (78). Nivolumab shows a dose-dependent toxicity signal, with 3 mg/kg weight-based dosing associated with higher AKI rates than 240 mg flat dosing (incidence 6.2% vs 4.1%, p=0.03) (79). Geographic variations exist: Asian populations report higher rates of severe nephritis (grade ≥3:2.8% vs 1.4% in Western cohorts),possibly reflecting HLA haplotype associations (HLA-DRB1*04:05 in Japanese populations) (80). Combination ipilimumab+nivolumab demonstrates synergistic toxicity (OR 4.2 for AKI vs nivolumab monotherapy), with sequence-dependent effects (ipi→nivo induction vs concurrent administration) (81). These findings underscore the need for drug-specific and population-adjusted risk stratification rather than class-level generalizations.
